# Endothelial Progenitor Cells of the Human Placenta and Fetoplacental Circulation: A Potential Link to Fetal, Neonatal, and Long-term Health

**DOI:** 10.3389/fped.2017.00041

**Published:** 2017-03-15

**Authors:** Diane L. Gumina, Emily J. Su

**Affiliations:** ^1^Obstetrics and Gynecology, University of Colorado School of Medicine, Aurora, CO, USA

**Keywords:** endothelial progenitor cells, umbilical cord blood, placenta, endothelial colony-forming cells, circulating progenitor cells

## Abstract

The fetoplacental circulation plays a key role in both short- and long-term outcomes, and aberrant flow indices as manifested by abnormal fetal Doppler velocimetry within this compartment have been associated with significant adverse consequences. These include fetal growth restriction, which often coexists with preeclampsia, and long-lasting medical issues as a result of both the underlying pathology and prematurity such as bronchopulmonary dysplasia, chronic lung disease, and neurodevelopmental delay. Furthermore, it is also clear that exposure to an abnormal *in utero* environment increases risk for long-term, adulthood issues such as cardiovascular disease. Endothelial progenitor cells (EPCs) have been implicated in vasculogenesis and angiogenesis, and they have been isolated from both human placenta and umbilical cord blood. This review outlines the extensive nomenclature of EPCs, summarizes existing literature surrounding human placental and umbilical cord blood EPCs, explores their potential role in pregnancy complications and adverse perinatal outcome, and highlights key areas where future investigations are needed.

## Introduction

Of the three main components that shape placental function—the maternal uteroplacental circulation, placental trophoblast, and fetoplacental blood flow—it is the fetoplacental circulation that has been clinically demonstrated to be most highly related to adverse perintal outcome. For instance, pregnancies complicated by fetal growth restriction (FGR) with abnormal fetal Doppler velocimetry (e.g., umbilical arteries, middle cerebral artery with concern for “brain-sparing,” and ductus venosus) are at significantly elevated risks for stillbirth and neonatal death (1, 2). Survivors are at higher risk for chronic medical problems and neurodevelopmental delay (1, 2). Furthermore, even if a growth-restricted fetus emerges from the perinatal and early childhood periods without adverse consequences, multiple lines of evidence suggest that they remain at increased risk for long-term, adulthood issues such as cardiovascular disease (3–5).

From a structural perspective, scanning electron microscopy, stereological analysis, and mathematical modeling suggest that FGR placentas demonstrate impaired placental vascular angiogenesis (6–8). Although this is just one condition in which the fetoplacental vasculature is impaired, it highlights the importance of this compartment in pregnancy and long-term outcome.

Recently, there has been substantial focus on endothelial progenitor cells (EPCs) and their role in vasculogenesis, angiogenesis, and even re-endothelialization of injured vessels. This field continues to evolve in many areas including nomenclature, methods of isolation and culture, and mechanistic roles during pathogenesis. EPCs have been isolated from the human placenta and umbilical cord blood, and in comparison to those derived from adult peripheral blood mononuclear cells (PBMNCs), demonstrate unique features such as enhanced proliferative and clonogenic potential. This suggests that placental and/or cord blood EPCs might play a role in development of the fetoplacental vasculature, and thus, may be potential targets for treatment modalities aimed at improving pregnancy, fetal, neonatal, and long-term outcomes.

## EPC History

The initial discovery of a population of putative EPCs from adult peripheral blood in the late 1990s (9) changed two widely accepted paradigms. First, it challenged the existing notion that vasculogenesis occurs only during fetal development. Second, it disputed the concept that angiogenesis in adults is only able to arise through extension of mature vascular endothelium. Since this initial discovery, much has been uncovered about the function and classification of EPCs. In this review, we discuss the current nomenclature, history of various sub-populations, EPCs isolated from both umbilical cord blood and placenta, and the association of EPCs with adverse pregnancy outcomes.

Isolation of an EPC population was first performed by Asahara and colleagues (9). However, the identification method used to isolate these cells did not include a unique identifier specific to EPCs. As such, many groups have since worked to further characterize and develop a method to unequivocally identify EPCs. Unfortunately, a universal, indisputable approach to ascertaining a progenitor population has yet to be found. Progress, however, has been made to further characterize EPCs and EPC sub-types, and along the way, new nomenclature and identification techniques have been introduced. Differing names and techniques can be confusing and makes it difficult to decipher if previous reports are applicable to current investigation. To best understand the current state of the field, it is helpful to have an appreciation for where the field began.

The initial identification of EPCs utilized Ficoll centrifugation of peripheral blood to obtain a mononuclear cell population, and within this population, either CD34^+^ or Flk-1^+^ (also known as vascular endothelial growth factor receptor 2 or kinase insert domain receptor) cells were isolated with magnetic beads coated with the respective antibodies. These two antigens were individually targeted because both are expressed by hematopoietic stem cells prior to differentiation. Enriched cells were plated under various conditions. Attached CD34^+^ cells after 7 days of culture appeared spindle-like and expressed endothelial-specific markers including *Ulex europaeus* agglutinin-1, factor VIII, CD31, endothelial nitric oxide synthase, and DiI-labeled acetylated LDL. They also demonstrated an endothelial cell-like phenotype, with the ability to produce nitric oxide in response to acetylcholine and vascular endothelial growth factor. *In vivo*, these cells were incorporated into foci of neovascularization in a rabbit model of unilateral hindlimb ischemia. In total, these investigators concluded that PBMNCs isolated with anti-CD34 or anti-Flk-1 were able to differentiate into endothelial cells, and this method of isolation and identification became the standard for assessing EPCs.

## EPC Isolation *via* Cell Culture

**Figure 1:**
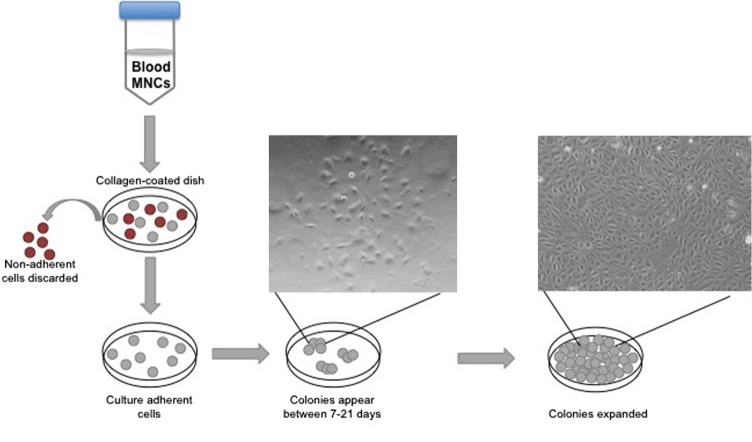
**Isolation of endothelial colony-forming cell schematic**. The diagram depicts peripheral blood mononuclear cell separation *via* a Ficoll gradient, plating on collagen, and the appearance of colonies with cobblestone morphology.

**Figure 2:**
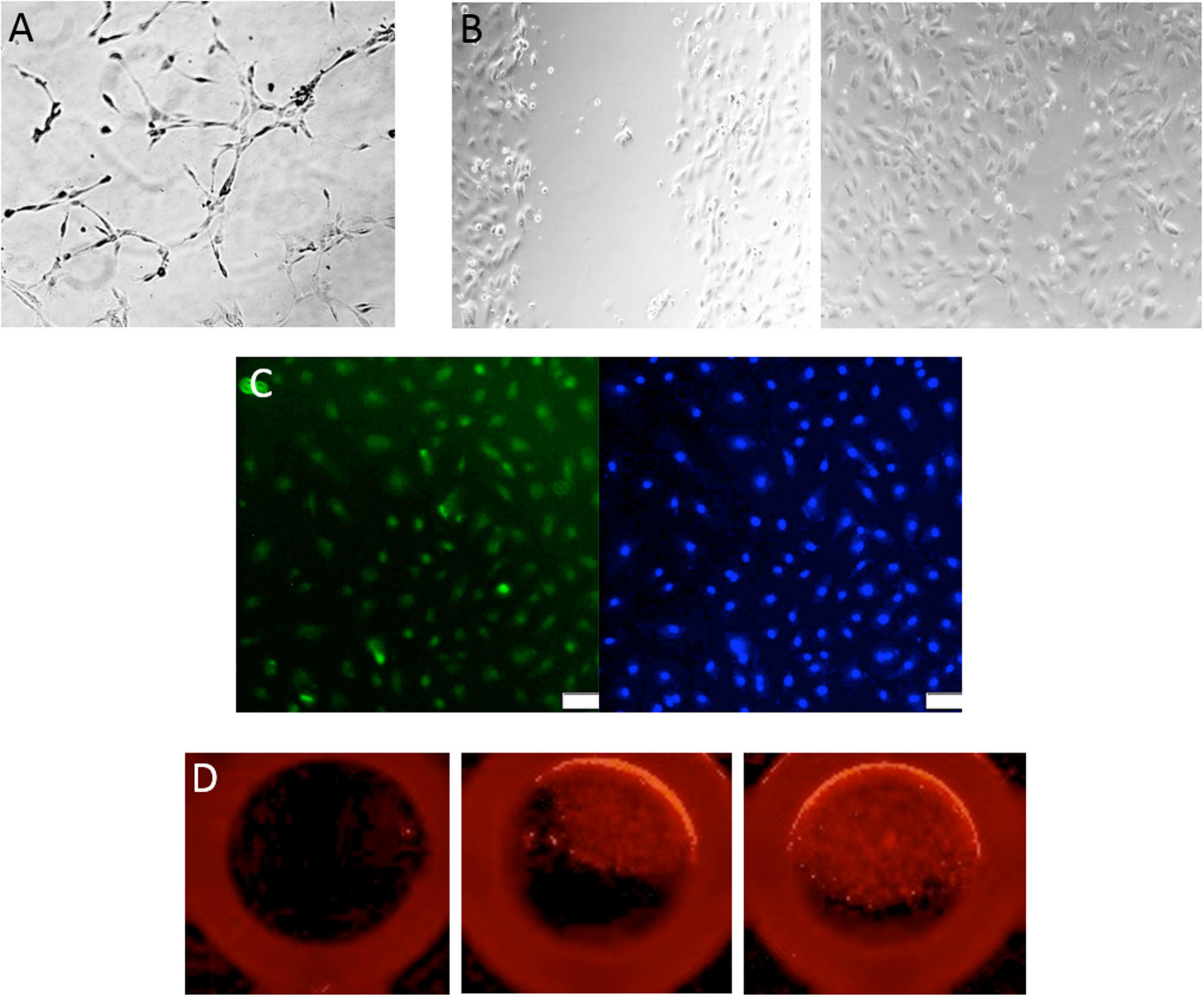
**Assessment of endothelial colony-forming cell (ECFC) function**. **(A)** Representative image of a tube formation assay, where ECFCs are capable of forming capillary-like structures with the formation of branches and closed loops. **(B)** A classic wound migration demonstrates that ECFCs are able to migrate and close the wound. **(C)** ECFC proliferation is shown with BrdU staining in green (DAPI in blue). **(D)** Single-cell assay shows that ECFCs are capable of repopulation from a single cell.

Different cell culture methods of isolating EPCs utilize principles from the culture conditions as mentioned above, with each technique resulting in the isolation of different cell types. The method isolating endothelial colony-forming cells (ECFCs) is widely accepted as the closest representation of an EPC *in vitro* population. Isolation of ECFCs includes Ficoll centrifugation of PBMNCs, plating on collagen-1 coated plates, culturing in complete EGM-2 media (cEGM-2; Lonza) with 20% FBS, and expansion of colonies that appear typically between 14 and 21 days after isolation (Figure 1). This method produces colonies with cobblestone morphology indicative of an endothelial cell type. Functionally, these cells are able to migrate and form capillary-like structures, proliferate, and repopulate from a single cell (Figure 2) (10). These cells can sustain multiple passages in culture, but most experts recommend use of low passage number cells (P2–5) for experimental purposes. This cell type is also known as late outgrowth EPCs, blood outgrowth endothelial cells (10, 11), and can be defined as low or high proliferative potential (LPP and HPP, respectively) (12).

While this method is accepted, it has limitations. Currently, there are no studies linking ECFCs, an *in vitro* population, to physiologic cell populations. Another issue with this method is the quantity of blood required to produce colonies. Estes et al. has recommended a minimum of 16 mL of peripheral blood for the isolation of ECFCs in healthy adults (13). When isolating from umbilical venous cord blood, which has a higher percentage of ECFCs than adult peripheral blood (12), a minimum of 5 mL is required, although 10–20 mL is recommended. However, in conditions where the pregnancy is affected by certain pathologies, especially those resulting in preterm delivery, collecting an appropriate amount of cord blood can be difficult. Obtaining an adequate amount can also become problematic when trying to isolate ECFCs from infants and children.

Other cell culture isolation methods have been reported in the literature including colony-forming unit-endothelial cells (CFU-ECs) (14) and CFU-Hill (15) and early outgrowth EPC. A description of the nomenclature, isolation method, and limitations are outlined in Table 1. All of these names refer to similar cells, in that they have a spindle-like morphology, do not incorporate into vessels *in vivo*, and are likely of myeloid or lymphoid progenitor background (10, 16, 17).

**Table 1:** **The most commonly used nomenclature, isolation method, and associated identification markers of endothelial progenitor cells (EPCs) *in vitro***.

Cell culture models				

Name	Other names	Isolation method	Identifying markers	Reference
Endothelial colony-forming cells	Blood outgrowth endothelial cells Late outgrowth EPCs	Peripheral blood mononuclear cells (PBMNCs) are isolated from peripheral blood with a Ficoll gradient. Cells are plated on collagen-1, grown in cEGM-2, and colonies appear between 14 and 21 days in culture	Expression of CD31; CD141; CD105; CD146; CD144; vWF; Flk-1; CD34; CD133; CD117; eNOS	(12, 18)
			Negative staining for CD45, CD14	
			Single-cell assay: able to repopulate from a single cell	
			Able to form capillary structures *in vitro*	
			Cobblestone morphology	
Colony-forming unit-endothelial cells	Early outgrowth EPCs, CFU-Hill EPCs	PBMNCs are isolated from peripheral blood similar to above, plated on fibronectin, grown in M199 medium, and colonies appear between 5 and 7 days in culture	Expression of CD34, vWF, CD144, Flk-1, UEA-1, Tie-2	(10, 14, 19)
			DiI-Ac-LDL uptake	
			Negative staining for VCAM-1	
			Spindle-like morphology	

*vWF, von Willebrand factor; Flk-1, vascular endothelial growth factor receptor-2; eNOS, endothelial nitric oxide synthase; UEA-1, Ulex europaeus agglutinin-1; VCAM-1, vascular cell adhesion molecule-1; Tie-2, TEK receptor tyrosine kinase; DiI-Ac-LDL, DiI-conjugated acetylated low-density lipoprotein*.

## EPC Isolation *via* Flow Cytometry

**Figure 3:**
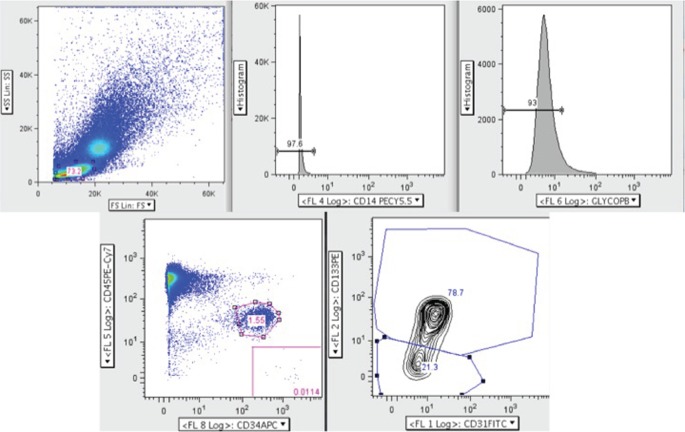
**Circulating progenitor cell (CPC) flow cytometry gating strategy adapted from Gumina et al. (23)**. Here, peripheral blood mononuclear cells were analyzed by polychromatic flow cytometry. Live mononuclear cells were selected and then gated for CD14^−^ and glycophorin A^−^ cells to exclude erythrocytes and macrophages. Next, the CD45^dim^ and CD34^+^ population was selected from which the pro-angiogenic (CD45^dim^ CD34^+^ CD31^+^ AC133^+^) and non-angiogenic (CD45^dim^ CD34^+^ CD31^+^ AC133^−^) CPCs were identified.

Another commonly used method for identifying EPCs is flow cytometry, and similar to cell culture methods of isolation, there are many differing sets of antigens used. The most recent version identifies circulating progenitor cells (CPCs) and allows for sub-group analysis of pro-angiogenic CPCs (CD45^dim^CD34^+^CD31^+^AC133^+^) and non-angiogenic CPCs (CD45^dim^CD34^+^CD31^+^AC133^−^) (Figure 3) (13, 20). The current limitations of this method include at least 1 × 10^6^ cells for staining. This is not an issue when performed alone, but when paired with cell culture isolation, cell quantity may be inadequate. Additionally, as there are steps required for multiple antigen staining, these cells need to be fixed, and therefore it is not possible to culture these cells for ECFC comparison. It is important to note that ECFCs exist in the flow cytometry literature as well but are distinct from CPC populations. Specifically, ECFCs are a rare population thought to be identifiable with the CPC staining profile but instead of CD45^dim^, they are CD45^−^ (12, 21). Older methods using a combination of CD34^+^Flk-1^+^CD133^+^ are likely incorporating angiogenic macrophages (22), which confound much of the older literature. Table 2 provides a description of the nomenclature and staining protocols in the literature.

**Table 2:** **The most commonly used nomenclature and associated staining protocol for flow cytometry identification of endothelial progenitor cells (EPCs)**.

Flow cytometry		

Name	Staining protocol	Reference
EPC/circulating endothelial precursor	CD45^−^ and/or CD34^+^AC133^+^KDR^+^	(9, 24)
Circulating endothelial cell	CD31^bright^CD34^+^CD45^−^CD133^−^	(25)
Pro-angiogenic circulating progenitor cell (CPC)	CD45^dim^CD34^+^CD31^+^AC133^+^	(13, 20)
Non-angiogenic CPC	CD45^dim^CD34^+^CD31^+^AC133^−^	(13, 20)
Endothelial colony-forming cell	CD45^−^CD34^+^CD31^+^AC133^−^CD146^+^	(13, 21)

## Placental and Umbilical Cord Blood EPCs

There has been considerable interest in alternative sources for EPCs beyond adult peripheral blood or bone marrow isolation. Various groups have demonstrated the presence of EPCs within both the human placenta and umbilical cord blood. Investigation of EPCs within the placenta has been limited, while literature surrounding cord blood EPCs has been much more robust. Nevertheless, a few different laboratories have described isolation of ECFCs from the human placenta, although some differences exist such as surface molecule expression (26–28). For example, Sölder et al. isolated CD45^−^CD34^+^CD133^+^Flk-1^+^ fetal endothelial cells from the placenta and showed that they were able to form tubes on a Matrigel assay (28). By contrast, CD45^−^CD34^−^CD31^+^Flk-1^+^CD144^+^ cells were isolated by Rapp and colleagues, and these cells were able to form chimeric blood vessels in an *in vivo* vasculogenesis bioassay (26).

Unlike placentally derived EPCs, umbilical cord blood EPCs have been more extensively investigated. Earlier studies isolated EPCs that were characterized *via* early methodologies first described by Asahara and colleagues (9), which again, was the first description of a putative EPC population in adult peripheral blood (29, 30). Thus, these early cord blood EPCs are more consistent with CFU-ECs and not ECFCs. More recent studies of cord blood ECFCs demonstrate expression of various endothelial-derived surface markers (Table 1), with these cells exhibiting significant clonogenic and proliferative potential (12, 31). Importantly, cord blood ECFCs are enriched and display enhanced clonogenic and proliferative potential in comparison to adult peripheral blood (12). However, adult peripheral and cord blood ECFCs do not show any difference in tube formation capability or induction of vascular cell adhesion molecule-1 with inflammatory stimuli (12). When compared to placental ECFCs, though, cord blood ECFCs form significant fewer blood vessels in an *in vivo* vasculogenesis assay (26).

## Umbilical Cord Blood EPCs and Disease

In spite of the current limitations in identifying EPCs, there are numerous studies that have indicated reduced colony number and/or dysfunction of EPCs isolated from the umbilical cord blood of pregnancies complicated by preeclampsia, FGR, and gestational diabetes mellitus (GDM). In this review, we focus solely on studies that analyze either ECFCs in cell culture or CPCs derived from flow cytometry in order to avoid further confusion with other isolation methods that likely produce cell types independent of EPCs. It is important to note that other literature exists analyzing other cell populations described in Tables 1 and 2.

With regard to preeclampsia, different groups of investigators have demonstrated both reductions in circulating number and abnormal function of ECFCs isolated from the venous cord blood of babies born to preeclamptic mothers (23, 32, 33). Specifically, Gumina et al. showed a decrease in both pro- and non-angiogenic subsets of CPCs identifiable by flow cytometry in pregnancies complicated by preeclampsia in comparison to normotensive controls. All three reports also indicate fewer ECFC colony numbers in their respective preeclamptic populations. From a functional perspective, two groups reported that ECFCs from cord blood of preeclamptic pregnancies demonstrated diminished growth and migration (23, 32), while other laboratories found no difference in ECFC tube formation (23, 33). However, von Versen-Höynck et al. demonstrated a deficiency in preeclamptic ECFC tube formation, with partial improvement with vitamin D3 treatment (32).

Similar findings have been demonstrated in pregnancies complicated by FGR. Cord blood from FGR-complicated pregnancies showed fewer CPCs and ECFCs in comparison to controls, although this was seen only in arterial cord blood (34). ECFCs from the FGR offspring also showed diminished proliferation and migration. Furthermore, FGR ECFCs implanted into mice prepared for an *in vivo* vasculogenesis bioassay resulted in a sixfold increase in *de novo* capillary formation in comparison to controls (34). Taken together, the abnormalities seen in cord blood ECFCs in preeclampsia and FGR may be one mechanism that contributes to placental dysfunction and long-term elevated risks for cardiovascular disease in these offspring.

There is conflicting data regarding CPCs and ECFCs from the cord blood of GDM pregnancies. For example, one group of investigators found a decrease in CPCs and the CPC:non-CPC ratio in cord blood from GDM pregnancies in comparison to controls, but there was no difference in ECFCs, suggesting that endothelial function is intact at birth (35). By contrast, others have shown a decrease in ECFC colonies, proliferation, migration, and tube formation in cord blood of pregnancies complicated by GDM (36). From a mechanistic perspective, fetal ECFCs exposed to *in vitro* hyperglycemia demonstrated impaired migration and diminished tube formation in comparison to those exposed to normoglycemic conditions (36). ECFCs from GDM pregnancies, however, were also found to be resistant to hyperglycemia-induced senescence (36, 37). In total, this suggests that although cord blood EPCs in GDM pregnancies may have undergone a phenotypic alteration that renders them tolerant to a hyperglycemic environment, they still demonstrate functional abnormalities that may contribute to the increased risks of cardiovascular disease in offspring of diabetic women.

## Current Limitations and Future Areas of Investigation

There are several limitations within the field that may amplify discrepancies between findings in different studies. First, a comprehensive characterization of ECFCs in relation to normal physiology of the fetus and neonate is lacking. Additionally, knowledge of gestational age norms is also essentially non-existent, and this further hampers the field of investigation regarding EPCs and other pathogenic conditions that relate more directly to impaired placental vascularization, including FGR and stillbirth. Second, there are methodological issues that have yet to be standardized. For example, when ECFC number is assessed, it refers to the number of colonies that appear. ECFC colonies typically arise between 14 and 21 days *in vitro*, although colonies can still develop beyond this time frame (33). Thus, discrepancies in the literature may be a result of when the colonies are counted, and this may be one reason why studies differ in their interpretation of how ECFCs are specifically impacted by each condition. Third, controversy also exists when assessing ECFC function. Functionality is most commonly evaluated by measures of proliferation, migration, and ability to form capillary-like structures. As discussed above, all of these cellular processes can be assayed with various techniques and each technique can have slight differences that result in differing findings. Additionally, few studies incorporate *in vivo* models such as ischemic injury animal models in which a Matrigel plug embedded with patient-derived ECFCs is injected into the area of ischemia and ECFC incorporation into newly formed vessels is later analyzed. This model (38, 39) would yield a better understanding of ECFC function in a physiological setting.

In addition to methodological issues, it is also possible that different study populations are being investigated. For example, preeclampsia can present across a wide gestational age range and with varying degrees of severity. However, it has been shown that ECFCs are enriched at different gestational ages within umbilical cord blood, with gestational age likely to affect findings (31). Furthermore, the effect of the severity of the condition itself on ECFCs has also not yet been explored. Because number and function of ECFCs has been associated with adverse neonatal outcomes such as moderate or severe bronchopulmonary dysplasia, which itself has also been linked to severity of preeclampsia and FGR, it is not inconceivable that the status of the disease may affect ECFCs (18). As another example, ECFCs are increased in infants affected by chorioamnionitis, further suggesting that *in utero* environment may play a role on umbilical cord blood and placental EPCs (18).

Finally, in addition to continued cord blood EPC research, further investigation is also needed with regard to placental EPCs. The few existing studies utilize different isolation methods, demonstrate slight differences in immunophenotype, and perhaps most compellingly, suggest that there might be enhanced colony formation and functional characteristics in comparison to umbilical cord blood EPCs. As the field continues to advance, umbilical cord blood and placental EPCs are areas ripe with opportunity to better understand mechanisms underlying pregnancy-related diseases and adverse perinatal outcome. Continued investigation may yield preventative treatments or interventions for these pregnancy and perinatal complications in the future. Yet, this field has the potential to provide treatment targets beyond perinatal and neonatal outcomes by further elucidating mechanisms of fetal programming effects that contribute to increased risks for disease later in life.

## Author Contributions

Both individuals actively contributed to the organization, intellectual content, and writing of this review.

## Conflict of Interest Statement

The authors declare that the research was conducted in the absence of any commercial or financial relationships that could be construed as a potential conflict of interest. The reviewer AJ and handling Editor declared their shared affiliation, and the handling Editor states that the process nevertheless met the standards of a fair and objective review.
